# Cellular Disulfide Bond Formation in Bioactive Peptides and Proteins

**DOI:** 10.3390/ijms16011791

**Published:** 2015-01-14

**Authors:** Nitin A. Patil, Julien Tailhades, Richard Anthony Hughes, Frances Separovic, John D. Wade, Mohammed Akhter Hossain

**Affiliations:** 1 Florey Institute of Neuroscience and Mental Health, the University of Melbourne, Victoria 3010, Australia; E-Mails: nitin.patil@florey.edu.au (N.A.P.); julien.tailhades@florey.edu.au (J.T.); john.wade@florey.edu.au (J.D.W.); 2 School of Chemistry, the University of Melbourne, Victoria 3010, Australia; E-Mail: fs@unimelb.edu.au; 3 Department of Pharmacology and Therapeutics, the University of Melbourne, Victoria 3010, Australia; E-Mail: rahughes@unimelb.edu.au; 4 Florey Departments of Neuroscience and Mental Health, the University of Melbourne, Victoria 3010, Australia

**Keywords:** bioactive peptides, disulfide bonds, peptide and protein folding, oxidative folding, recombinant technology

## Abstract

Bioactive peptides play important roles in metabolic regulation and modulation and many are used as therapeutics. These peptides often possess disulfide bonds, which are important for their structure, function and stability. A systematic network of enzymes—a disulfide bond generating enzyme, a disulfide bond donor enzyme and a redox cofactor—that function inside the cell dictates the formation and maintenance of disulfide bonds. The main pathways that catalyze disulfide bond formation in peptides and proteins in prokaryotes and eukaryotes are remarkably similar and share several mechanistic features. This review summarizes the formation of disulfide bonds in peptides and proteins by cellular and recombinant machinery.

## 1. Introduction

Disulfide bonds are common structural motifs in many bioactive peptides and proteins including hormones, neurotransmitters, growth factors, enzyme inhibitors, and antimicrobial peptides [1,2,3]. They play a critical role in maintaining the overall fold of the peptides and proteins and are thereby often important for the function and stability of proteins and peptides. In nature, such bonds are usually formed during the posttranslational modification stage with the assistance of appropriate enzymes and co-factors whereas, in the laboratory, disulfide bonds in native proteins can be formed randomly in basic buffer via air oxidation or by regioselective methods. This review discusses biological means of disulfide bond formation in biological systems.

## 2. Folding of Peptides and Proteins in Normal Cells

**Figure 1:**
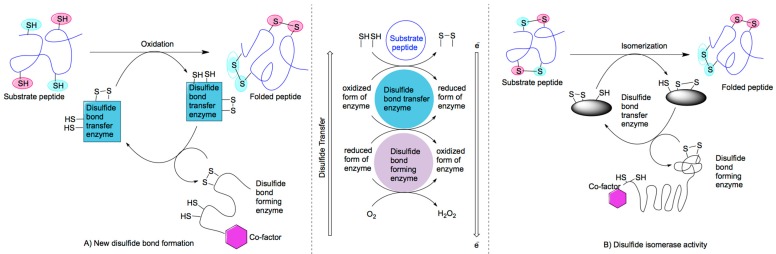
Schematic representation of general mechanisms by which a disulfide bond is formed by an intra-cellular enzyme-cofactor system: (**A**) Formation of new disulfide bonds; and (**B**) reshuffling of existing bonds by isomerase activity.

Cysteines, sulfhydryl-containing amino acids, which are located an appropriate distance or next to one another within a polypeptide chain, will form a disulfide bond through their oxidisable thiol groups. This bond will impart a fold in the chain of the protein or bend in its structure. Disulfide bond formation and its effect on protein folding has been a subject of great interest for at least half a century [4]. The first reported study by Anfinsen in 1973 revealed that disulfide bond formation inside the cell is spontaneous and that the amino acid sequence is sufficient to determine correct folding of the peptide or protein [4,5]. It was subsequently shown that there are several active disulfide bond-promoting enzymes and cofactors functioning in the cell [6,7] meaning that disulfide bonds are usually formed by a systematic network of intracellular enzymes [8]. These enzyme systems form a new disulfide bond or reshuffle the existing mispaired disulfide bond in substrate peptides (Figure 1).

Typically, these systems consist of a disulfide bond generating enzyme, a disulfide bond donor enzyme and a redox cofactor [9]. Interestingly, while these enzymes have very low homology, their functional motifs differ only marginally [10]. A minimum assembly requires C–(X)_n_–C (where X = amino acid; *n* = 1–3) motif in the active site associated with a redox co-factor. In some periplasmic enzyme systems, an arginine residue has been observed to stabilize the charge transfer complex between the cysteine and co-factor [11,12,13].

### 2.1. Mechanisms of Disulfide Bond Formation

**Figure 2:**
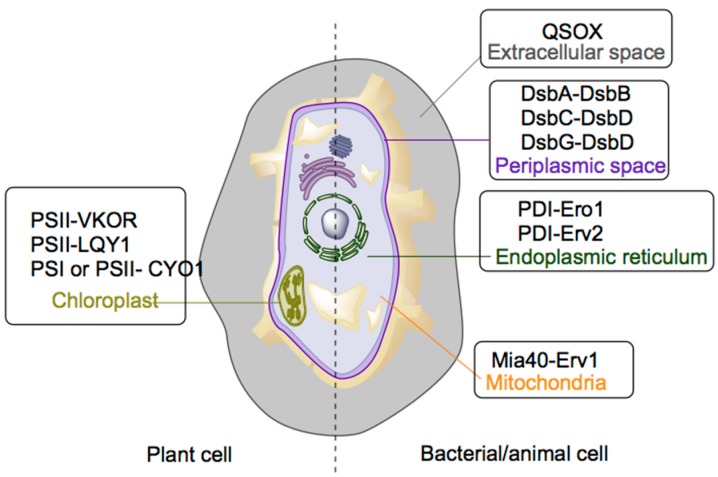
Cellular representation of enzyme systems and respective organelles.

The formation of disulfide bonds in bacterial (prokaryotic) cells is well characterized [14,15]. Generally, bacterial proteins are synthesized by ribosomal mRNA translation and disulfide bonds are subsequently formed as posttranslational modifications catalyzed by various enzymes located in the periplasm [16] or cytoplasm [17,18]. In higher animals the same process is performed in specific cell organelles, such as mitochondria, the endoplasmic reticulum (ER) and chloroplasts (Figure 2, Table 1).

**Table 1:** Cellular compartments and enzyme systems for disulfide bond formation.

Site	Disulfide Bond Transferring Enzyme	Disulfide Bond Generating Enzyme	Cofactor
Prokaryotic	DsbA	DsbB	Ubiquinone
Periplasm	DsbC	DsbD	Ubiquinone
	DsbG	DsbD	-
Endoplasmic reticulum	PDI	Ero1	FAD
	PDI	Erv2	FAD
Mitochondria	Mia40	Erv1	FAD
Chloroplast	PSII	LTO1	Phylloquinone or Hydroquinone
	PSII	LQY1	Zn (believed to be a cofactor)
	PSI and PSII	CYO1	Zn (believed to be a cofactor)
Extracellular space	QSOX	QSOX	FAD

#### 2.1.1. Periplasmic System

In prokaryotic cells, disulfide bond formation predominantly occurs through a network of periplasmic enzymes, the thiol-disulfide oxidoreductase family, called disulfide bond forming enzymes (Dsb) [19,20]. A series of disulfide oxidoreductase enzymes, including DsbA, DsbB, DsbC and DsbD, have been identified over the last 25 years. Elucidation of the crystal structure of *E. coli* DsbA enabled investigation into the mechanism of disulfide bond formation [21]. This enzyme system introduces a disulfide bond to a newly synthesized protein by means of DsbA-DsbB and ubiquinone (UQ) [22]. DsbA is the primary disulfide bond donor and its active state is the oxidized form with the Cys30–Cys33 disulfide bond. It is kept in the oxidized, active state by membrane bound protein, DsbB, which transmits electrons from DsbA to UQ. DsbB has been predicted to have two periplasmic loops and each of the loops contains one pair of essential cysteines: Cys41–Cys44 and Cys104–Cys130. While the Cys104–Cys130 pair is involved directly in the disulfide exchange with DsbA, the Cys41–Cys44 pair is the target of oxidation by UQ [13,23,24,25,26].

DsbA is known to have no proofreading activity and can form incorrect disulfides in proteins with multiple cysteines. These incorrect disulfide bonds are corrected by a protein disulfide isomerase, DsbC, which is kept in the reduced and active configuration by a membrane-bound protein, DsbD. The DsbC/DsbD isomerization pathway is considered to be isolated from the DsbA/DsbB pathway [27,28,29,30,31].

#### 2.1.2. Endoplasmic Reticulum System

In organisms such as fungi and mammals where protein folding is compartmentalized and complex, disulfide bond formation takes place in specialized organelles such as the ER and mitochondria [32]. The main reasons are: first, the cytosolic environment is reducing due to the high concentration of thioredoxin reductase and glutathione reductase, and second, the availability of supporting systems for appropriate protein folding for disulfide bond formation [33,34]. Ero-1 is the predominant disulfide bond-generating enzyme in ER and Erv2 principally in fungal cells [35]. The most studied transfer enzyme is protein disulfide isomerase (PDI) and the most studied enzyme system in such cells is “PDI-Ero1” [36].

Even though there is very poor sequence homology, the structural features of Ero-1 and Erv2 are similar to DsbB where one “C–(X)_n_–C” motif generates a disulfide bond together with FAD (flavin adenine dinucleotide) and another “C–(X)_n_–C” maintains unidirectional propagation of redox equivalents. Moreover, both these enzymes are associated with FAD where an isoalloxazine ring is embedded within the active site [37]. In Ero-1, the first cysteine pair is located in the “C–X–X–C–X–X–C” motif which generates a disulfide bond [38,39]. In the second cysteine pair, “C–(X)_n_–C”, the residues between two cysteines varies between paralogs of Ero-1 [40]. Similarly, the catalytic core of Erv2 has “C–X–X–C” and “C–(X)_n_–C” (where *n* = 1–4) motifs. However, there is no arginine residue as is found in the active site of the DsbB enzyme.

The mammalian ER contains two paralogs of Ero1, three Erv2-like proteins [35] and about twenty PDI family proteins [41]. Transfer of disulfide bonds is carried out by PDI. This oxidoreductase enzyme is found to play a role in isomerization as well as producing a disulfide bond in newly synthesized peptides and proteins. PDI contains two thioredoxin-like domains, a and a', each of which contains an active site within the CXXC motif [42].

#### 2.1.3. Mitochondria

The mitochondrial inter-membrane space (IMS) has a similar environment to that of the cytoplasm of mammalian (eukaryotic) cells. Thus, there is a special enzyme system working in the IMS of mitochondria. The sulfhydryl oxidase Erv1 is a disulfide bond generating enzyme and the redox dependent receptor Mia40 acts as transfer protein [43,44,45]. Disulfide bonds are introduced to the substrate protein via a “C–P–C” motif of Mia40 [46,47]. Mia40, also known as Tim40, was identified as a protein that mediates sorting of Tim proteins [48,49] and the folding of coiled-coil helix coiled-coil helix (CHCH) proteins [43], such as COX [50,51] in mitochondria [52,53]. Thus, it is important to maintain the Mia40 CPC motif in an oxidized form, which is ensured by sulfhydryl oxidase, Erv1 [54,55]. As with Ero1, Erv1 also contains FAD as a cofactor, which generates a disulfide bond between “C–X–X–C” motifs of Erv1 [56,57].

#### 2.1.4. Chloroplasts

Like other eukaryotic organisms, disulfide bond formation in plant cells is carried out in the ER [58,59], and mitochondria [60,61]. However being photosynthetic cells, they possess chloroplasts where there is a huge burden to fold hundreds of enzymes in order to maintain photosynthetic activity [62]. Thus, chloroplasts contain a specialized enzyme system for protein oxidation and folding [63,64].

Vitamin K epoxide reductases (VKOR) are members of a large family of enzymes that exist in a wide range of organisms including bacteria, archaea, vertebrates and plants [65]. Members of this family can mediate disulfide bonding via different mechanisms [66]. However, they all comprise a conserved “C–X–X–C” motif similar to DsbB enzymes in bacteria. This can be oxidized to generate a disulfide bond by transferring electrons to quinone [67]. Arabidopsis VKOR homolog known, as lumen thiol oxidoreductase1 (LTO1) is a plant homologue associated with a quinone moiety in thylakoid. LOT1 reduces quinone to generate a disulfide bond in the “C–X–X–C” motif, which is then transferred to a luminal subunit of Photosystem II (PSII) [68]. There is evidence of quinones such as phylloquinone (vitamin K) [69] and hydroquinone [70] playing roles in the redox cycle. However, the mechanism of electron transfer is not clearly understood.

Other known enzymes include low quantum yield of PSII1 (LQY1), a small Zn-finger protein involved in repair mechanisms by disulfide bond formation [71], and CYO1 (a Japanese word “shi-yo-u” which means cotyledon), an integral membrane protein of thylakoid associated with PSI and PSII [72]. Both of them possess a Zn finger motif [71,72], and are assumed to have PDI activity. However, a lack of structural information limits an understanding of the exact mechanism underlying the electron transfer.

#### 2.1.5. Extracellular Space

Quiescin sulfhydryl oxidase (QSOX) is one of the most important enzymes that carries out disulfide bond formation and protein folding in extracellular and subcellular spaces. QSOX binds a FAD cofactor [73] which enables the generation of disulfide bonds. It also contains thioredoxin [74] motifs which aid in transferring the disulfide to the substrate protein [75]. In mammals, cells secrete QSOXs into the extracellular space after modification by the Golgi complex [76]. Human QSOX1 has two thioredoxin domains, one of which contains a “C–X–X–C” motif similar to prokaryotic DsbA [74] and eukaryotic PDI [77].

## 3. Folding of Disulfide-Containing Bioactive Peptides and Proteins via Recombinant Technology

Recombinant DNA-mediated polypeptide production remains a popular approach for obtaining properly folded disulfide-containing protein and peptides. Many reviews are available that address the effect of various parameters such as different host organisms [78,79], expression vectors [80], expression rate [81,82], and purification [83,84]. With respect to host systems, many cell lines are available for expressing recombinant peptides and proteins such as *E. coli* [85], Chinese hamster ovary cells [86], human embryonic kidney cells [87], and *S. cerevisiae* [88]. The wide range of molecular biology options with *E. coli* systems provides many options to ensure proper disulfide bond generation [89,90]. *E. coli* expression is fast and inexpensive to scale-up but not all proteins are amenable to expression in this system [91,92].

A major challenge in obtaining a recombinant protein is correct folding. A protein synthesized in a recombinant cell is often produced in reduced form, which is then oxidized (refolded) using suitable folding conditions [93,94]. The target crude peptide/protein can be obtained in reduced form and then refolded with the aid of various enzymes as disulfide catalyst systems [95,96]. Alternatively, oxidative folding is possible without the use of any enzyme [97,98]. However, such refolding can be a problematic step. Misfolding is a major issue with cysteine-containing polypeptides resulting in low yields. This limitation has necessitated the development of new methodologies to enable the acquisition of correctly folded protein or peptide in its native form in high yields. To assist the refolding, various tactics have been employed including inclusion body formation [99,100], co-expression of supporting enzymes [101,102] and chemical assistance [100].

### 3.1. Co-Expressing Supporting Enzymes/Peptides

In a recombinant DNA cell, a nascent polypeptide is produced by translation. It is then either folded in the cytoplasm or translocated to a suitable oxidative environment such as the bacterial periplasm. Disrupting reducing pathways in the cytoplasm has been shown to allow disulfide bond formation in the cytoplasm of *E. coli* [103]. Furthermore, co-expression of Dsb enzymes was found to improve the yield of desired folded protein [89]. These approaches have been extensively used in the expression of disulfide-containing proteins such as chitinase [104], endopolygalcturonase, [105] and anti-freeze proteins [106]. Overexpression of thioredoxin has been shown to act as an oxidant, helping to maintain oxidative conditions in the cytoplasm [107] and resulting in improved yields of folded proteins [108,109].

The most common practice for expressing disulfide-containing proteins in bacterial hosts is to “hijack” the membrane translocation machinery and secrete the polypeptide after translation [110]. A precursor peptide is produced which can be translocated and then folded into its active form. In this case, peptide oxidative folding takes place conveniently but the crucial barrier is the translocation of nascent peptide into the periplasm. A leader peptide sequence is essential for this purpose. The correlation between hydrophobicity of the leader peptide and export mechanism can be a more efficient means of translocating the precursor peptide [111]. A leader peptide could be an endogenous signal peptide sequence [112,113,114], a phage-pIII leader peptide [115] or even a designed synthetic sequence [116]. Another more convenient tactic is the folding of a precursor peptide assisted by co-expression of disulfide bond forming enzymes [117], foldases [118] or even periplasmic chaperones [119].

Although continuous optimization has led to improved yields of expressed and folded protein, there are many more challenges in expressing multiple enzymes/peptides in a recombinant system. These include nonspecific binding of chaperones [120,121], incorrect folding of soluble target protein [122] or direct excretion of translation product into the culture medium [123,124]. Thus at times it can be advantageous to use chemicals to assist the folding of recombinantly-produced peptides.

### 3.2. Chemical Assistance in Recombinant Folding

Bearing in mind that the main obstacle to the expression of cysteine-containing recombinant proteins is a pronounced tendency to aggregate [125], low molecular weight chaperones have been shown to aid the folding of target proteins either by assisting cellular enzymatic systems or acting as independent chaperones [126,127]. Examples of this approach include the folding of immunotoxins by glycine betaine [128], cytochrome by sucrose [129], and proinsulin by l-arginine [130]. Recent studies have also shown that small molecules can be used to improve yields of recombinant folded peptides [131,132,133]. These include urea and lithium chloride which can minimize the extent of side products generated during oxidative folding [134]. The use of glutathione ester instead of glutathione contributed to improved folding ability of egg lysozyme [135]. Water-soluble reagents such as selenoxides have been successfully used in synthetic as well as recombinant peptides and protein [136].

## 4. Conclusions

Disulfide bond formation is critical for the proper folding of bioactive peptides and proteins. The increasing understanding of the intricate, complex *in vivo* disulfide bond forming process is providing important insights into the mechanisms of diseases that are caused by protein misfolding and may contribute to the development of corrective measures with therapeutic applications. This review aims to provide readers with comprehensive details and updated knowledge on how the disulfide network is formed and maintained in biological systems.
